# Long-acting protein drugs for the treatment of ocular diseases

**DOI:** 10.1038/ncomms14837

**Published:** 2017-03-23

**Authors:** Joy G. Ghosh, Andrew A. Nguyen, Chad E. Bigelow, Stephen Poor, Yubin Qiu, Nalini Rangaswamy, Richard Ornberg, Brittany Jackson, Howard Mak, Tucker Ezell, Vania Kenanova, Elisa de la Cruz, Ana Carrion, Bijan Etemad-Gilbertson, Roxana Garcia Caro, Kan Zhu, Vinney George, Jirong Bai, Radhika Sharma-Nahar, Siyuan Shen, Yiqin Wang, Kulandayan K. Subramanian, Elizabeth Fassbender, Michael Maker, Shawn Hanks, Joanna Vrouvlianis, Barrett Leehy, Debby Long, Melissa Prentiss, Viral Kansara, Bruce Jaffee, Thaddeus P. Dryja, Michael Roguska

**Affiliations:** 1 Novartis Institutes for BioMedical Research, Cambridge, Massachusetts 02139, USA

## Abstract

Protein drugs that neutralize vascular endothelial growth factor (VEGF), such as aflibercept or ranibizumab, rescue vision in patients with retinal vascular diseases. Nonetheless, optimal visual outcomes require intraocular injections as frequently as every month. Here we report a method to extend the intravitreal half-life of protein drugs as an alternative to either encapsulation or chemical modifications with polymers. We combine a 97-amino-acid peptide of human origin that binds hyaluronan, a major macromolecular component of the eye's vitreous, with therapeutic antibodies and proteins. When administered to rabbit and monkey eyes, the half-life of the modified proteins is increased ∼3–4-fold relative to unmodified proteins. We further show that prototype long-acting anti-VEGF drugs (LAVAs) that include this peptide attenuate VEGF-induced retinal changes in animal models of neovascular retinal disease ∼3–4-fold longer than unmodified drugs. This approach has the potential to reduce the dosing frequency associated with retinal disease treatments.

## Abstract (web summary)

Retinal vascular disease treatments involve frequent pharmacological intraocular administrations. Here the authors present a method to increase the half-life of injected drugs by fusing these to a hyaluronan-binding peptide, which might lead to less frequent retinal disease treatments.

A feature of retinal neovascular diseases including neovascular age-related macular degeneration (AMD), diabetic macular oedema and retinal vein occlusion is that choroidal or retinal blood vessels are abnormally leaky due to a vascular endothelial growth factor (VEGF)-dependent increase in vascular permeability. The resulting retinal oedema impairs retinal function and decreases visual acuity. Clinical trials have demonstrated that these effects can be ameliorated with intravitreal injections of anti-VEGF drugs. Affected eyes vary in the frequency of doses required to eliminate or maximally reduce the oedema. For example, some eyes with neovascular AMD require doses every month and over 75% require doses at least every 3 months^1^. The frequent dosing is a significant burden for patients and physicians^2^. Real-world visual outcomes are generally inferior to outcomes achieved in pivotal trials^3^ in part due to suboptimal dosing frequency. Furthermore, each injection entails a small risk of endophthalmitis, uveitis, vitreous haemorrhage and other complications^4^. Thus, there is a need for intravitreal anti-VEGF therapies that can be delivered less frequently and yet provide the same or better vision than has been achieved in pivotal trials. We report here one approach to develop such a long-acting therapy.

Our strategy was to select a vitreous component to which a long-acting drug would weakly bind, thereby prolonging its residence time in the eye. We used three criteria to identify the optimal vitreal target. First, the concentration of the target must be sufficient to bind a clinically relevant amount of drug. For example, the clinical dose of ranibizumab is 0.5 mg per eye or ∼10 nmol per dose; hence, there must be at least 10 nmol of the vitreal target in the eye. Second, the target should have a low concentration in non-ocular tissues, especially blood, so that there would be minimal systemic retention and exposure. Third, the target should have a low turnover inside the eye so that target-mediated drug clearance would be minimal.

The vitreous of the eye is a matrix with collagen type II (150–250 μg ml^−1^ or ∼4–7 nmol per eye of the collagen monomer) and albumin (100 μg ml^−1^ or ∼6 nmol per eye)^5^ as the major protein components and hyaluronan (34–700 μg ml^−1^ or ∼340–7,000 nmol per eye of the disaccharide building block^6^) as the major carbohydrate constituent. Human collagen type II has 120 repeat units of Gly-Pro-*X* (where *X* can be any amino acid) that could potentially serve as the most abundant binding epitope. However, collagen type II has a compact triple-helix structure that significantly reduces the exposure of many of the repeat units and thus the available sites to which an intravitreal drug could bind. Based on the three-dimensional structure of collagen and its concentration in human vitreous, we estimate that a human eye would have only ∼2 nmol of binding sites on collagen, corresponding to a dose of ∼0.1 mg per eye of a Fab, assuming one Fab molecule per binding epitope. Albumin, an abundant blood protein, has been used as a binding target or fusion partner to prolong the systemic half-life of antibodies and proteins^7^, and it has a molar abundance close to that required for a Fab drug such as ranibizumab. However, albumin has a half-life in vitreous of only a few days^8^; thus, drugs with an affinity for it may have a rapid target-mediated clearance. Furthermore, binding to albumin has the potential disadvantage of mediating a long systemic half-life of a drug after it diffuses out of the eye into the blood.

Hyaluronan is a high-molecular-weight polysaccharide consisting of unbranched linear polymers of varying lengths of the disaccharide unit D-glucuronic acid plus D-*N*-acetylglucosamine^9^. Hyaluronan is naturally synthesized by a class of integral membrane proteins called hyaluronan synthases and it is degraded by a family of enzymes called hyaluronidases. In the human body it is at highest concentrations in the vitreous of the eye, in the synovial fluid and in the umbilical cord^10^. The median hyaluronan concentrations in human and cynomolgus monkey vitreous are ∼130 μg ml^−1^ (range: 34–700 μg ml^−1^)^6^ and ∼102 μg ml^−1^ (range: 89–125 μg ml^−1^), respectively. In contrast, rabbit vitreous only has ∼31 μg ml^−1^ hyaluronan (range: 14–52 μg ml^−1^)^9^. Hyaluronan in vitreous including human vitreous exists as polymers with a molecular weight over 1,000 kDa. Although the turnover of hyaluronan in human vitreous is not known, the turnover of hyaluronan in the rabbit vitreous has been shown to be low with a *t*_1/2_ of approximately 72 days^11^. In addition, hyaluronan concentration in human vitreous increases with age^6^, making it an ideal target for retaining therapeutic drugs in eye diseases of the elderly. Based on the vitreal concentration, the 384 Da size of the repeating disaccharide unit of hyaluronan, and the size of a Fab, the vitreous of a human eye would have a predicted carrying capacity of up to approximately 2 mg of a Fab, making hyaluronan suitable as a binding target for long-acting protein drugs delivered to the eye.

In this report, we demonstrate that fusing a hyaluronan-binding peptide (HABP) to protein drugs increases their ocular half-life and concomitant efficacy duration in rabbit and cynomolgus pharmacodynamic models of retinal vascular disease. Based on this data, we expect a similar extension in ocular retention will be achievable in patients with retinal vascular diseases enabling significantly reduced dosing frequency.

## Results

### Identification of peptides with an affinity for hyaluronan

**Figure 1:**
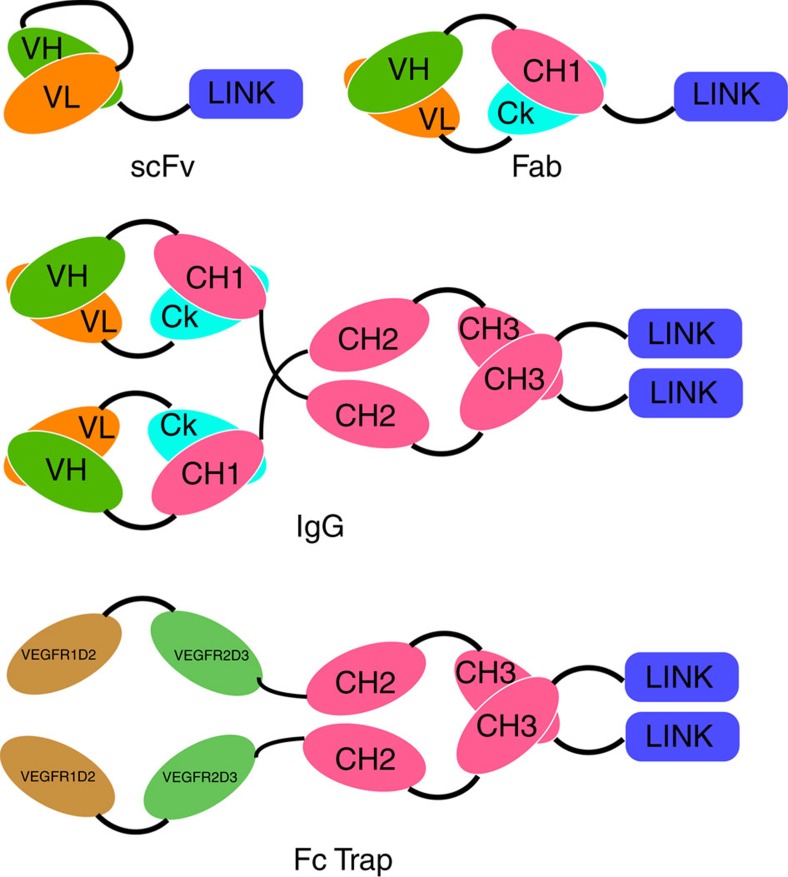
Schematic of LAVAs. The HA-binding peptide (HABP) was fused to the C-terminal end of scFvs, Fabs, Fc Traps and IgGs using a single GSGGG linker. The Fc traps and IgGs have two copies of the HABP, whereas the scFv and Fab have one copy.

Hyaladherins are proteins that contain one or a few copies of a conserved structural region called a LINK domain. The prototype hyaladherin is the human protein TSG6 (tumour necrosis factor (TNF)-stimulated gene 6 protein) encoded by the *TNFAIP6* gene (TNF α-induced protein 6). The LINK domain of TSG6 binds hyaluronan with an affinity (Kd) in the range of 100–1,000 nM (ref. 12). We created a series of 24 hybrid Fabs in which the LINK domains from 14 different hyaladherins (Supplementary Data 1) were individually fused to the carboxy terminus of the heavy chain of NVS0 (Fig. 1). NVS0 is the Fab version of brolucizumab, an anti-VEGF scFv currently in pivotal human clinical trials^13^. Nineteen of the fusion proteins could be expressed and purified in microgram to milligram quantities using a transient HEK293 expression method. We evaluated the ability of these hybrid Fabs to interact with hyaluronan polymers with an average molecular weight of 17 kDa using enzyme-linked immunosorbent assays (ELISA) or ForteBio Octet assays. Of the 19 hybrid Fabs, 13 had measurable binding to hyaluronan (Supplementary Data 1) and were further characterized.

### Fabs with HABPs have prolonged residence time in rat eyes

The 13 hybrid Fabs with measurable binding to hyaluronan, as well as an unmodified control Fab (ranibizumab), were radiolabelled with Iodine-121 and injected into the vitreous of rat eyes. The radioactivity in the eyes was measured non-invasively with a General Electric positron emission tomography (PET) instrument over a period of 10 days. The radioactivity of the labelled control Fab ranibizumab decreased rapidly and 48 h post injection only ∼0.9 % of the initial post-injection PET signal remained in the eye (Supplementary Data 1). No signal was detected 96 h post injection. In contrast, 9 of 11 hybrid Fabs had measurable radioactivity 96 h post injection, indicating ocular retention longer than ranibizumab. The hybrid Fab (NVS24) with the LINK domain from human TSG6 fused to NVS0 had the highest PET signal 96 h post injection, with about 13% of the signal remaining at that time point.

### Activity of LAVAs in VEGF-induced retinal leakage in rabbits

**Figure 2:**
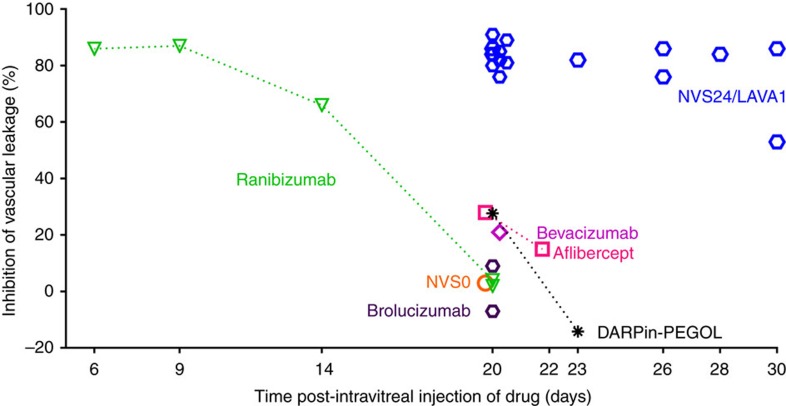
Duration of activity of anti-VEGF molecules in the rabbit retinal leakage model. Two days before fluorescein angiography, retinal vessel permeability was induced by an intravitreal injection of 10 pmol per eye hVEGF-A_165_. hVEGF-induced fluorescein leakage was measured in six to eight rabbit eyes for each anti-VEGF molecule and compared with control eyes that received intravitreal hVEGF but no anti-VEGF molecule. Each data point represents the average inhibition of vascular leakage compared with controls. LAVA1 inhibited fluorescein leakage when administered up to 28 days prior the hVEGF challenge (30 days before fluorescein angiography), whereas equimolar doses of ranibizumab, bevacizumab, aflibercept, brolucizumab or DARPin-PEGOL did not substantially inhibit fluorescein leakage when administered 18–21 days before the hVEGF challenge (20–23 days before fluorescein angiography). Data shown are from several independent studies.

To determine whether the nine hybrid Fabs that demonstrated long ocular retention in rat eyes had a long pharmacodynamic activity in a larger eye such as a rabbit eye, we assessed these hybrid Fabs in a retinal vascular leakage model we named the ‘rabbit VEGF-challenge' model. We first established what doses of recombinant human VEGF (specifically, the VEGF homodimer with each monomer having 165 amino acid residues, designated hVEGF-A_165_) injected into the vitreous of rabbit eyes disrupted the blood–retinal vessel barrier. The disruption of the blood–retinal barrier could be quantified by analysing images of the retinal vessels obtained during fluorescein angiography. An intravitreal dose of 400 ng (10 pmol) of hVEGF-A_165_ induced retinal leakage 48 h after injection. This leakage was inhibited by anti-VEGF molecules such as ranibizumab and aflibercept when they were dosed before the hVEGF-A_165_ injection (Fig. 2 and Supplementary Fig. 1). The approved clinical dose of ranibizumab (0.5 mg per eye or 10 nmol per dose) inhibited fluorescein leakage induced by 10 pmol of hVEGF-A_165_ for ∼30 days (Fig. 7). To enable more rapid screening, the time course of the rabbit model was shortened to 3 weeks by lowering the ranibizumab dose (the control comparator) to 5 μg per eye (105 pmol per dose). At this molar dose, ranibizumab and other control anti-VEGF drugs such as bevacizumab (an IgG), aflibercept (a receptor trap), NVS0 (a Fab) and brolucizumab (an scFv) did not substantially suppress hVEGF-A_165_-induced retinal vascular leakage 3 weeks post dosing. However, two of the nine hybrid Fabs, NVS8 and NVS24 suppressed VEGF-induced vascular leakage 3 weeks after dosing, with NVS24 producing the most substantial suppression. In additional independent studies, the activity of NVS24 persisted at least 4 weeks after a single dose (Fig. 2). The results indicated that fusion of a HABP did not interfere with the drug's ability to neutralize hVEGF in rabbit eyes *in vivo*, and that hybrid molecules had activity that persisted longer than non-modified anti-VEGF molecules. Owing to these properties, NVS24 was categorized as a long-acting anti-VEGF antibody or ‘LAVA'. It is hence referred to as LAVA1.

Upon completion of the final fluorescein angiography assessments in the pharmacodynamic experiments, rabbits were killed and eyes were enucleated to isolate viteous humor, to measure terminal drug levels (Table 1). The average amount of LAVA1 remaining at day 21 was 9.8 pmol per eye (∼10% of the injected dose), which was significantly higher than the amount of drug remaining 20 days after equimolar doses of either ranibizumab (0.1 pmol per eye or ∼0.1% of the injected dose) or aflibercept (2.1 pmol per eye or ∼2% of the injected dose). The average amount of NVS8 remaining at day 21 was 0.38 pmol per eye or ∼0.4% of the injected dose, which was significantly lower than that of LAVA1 and consistent with its reduced activity at 3 weeks. The terminal vitreal concentrations of the remaining LAVA molecules were similar to that of ranibizumab, consistent with their lack of efficacy at 3 weeks (Supplementary Data 1). Thirty-one days post dosing, 3 pmol per eye or ∼3% of the injected dose of LAVA1 was still present in rabbit eyes. The 80-fold higher terminal levels of LAVA1 21 days post injection compared with ranibizumab or aflibercept indicated that LAVA1 has a longer ocular half-life, consistent with its longer-lasting ability to neutralize VEGF in rabbit vitreous.

### LAVA1 inhibits laser-induced CNV in cynomolgus monkeys

**Figure 3:**
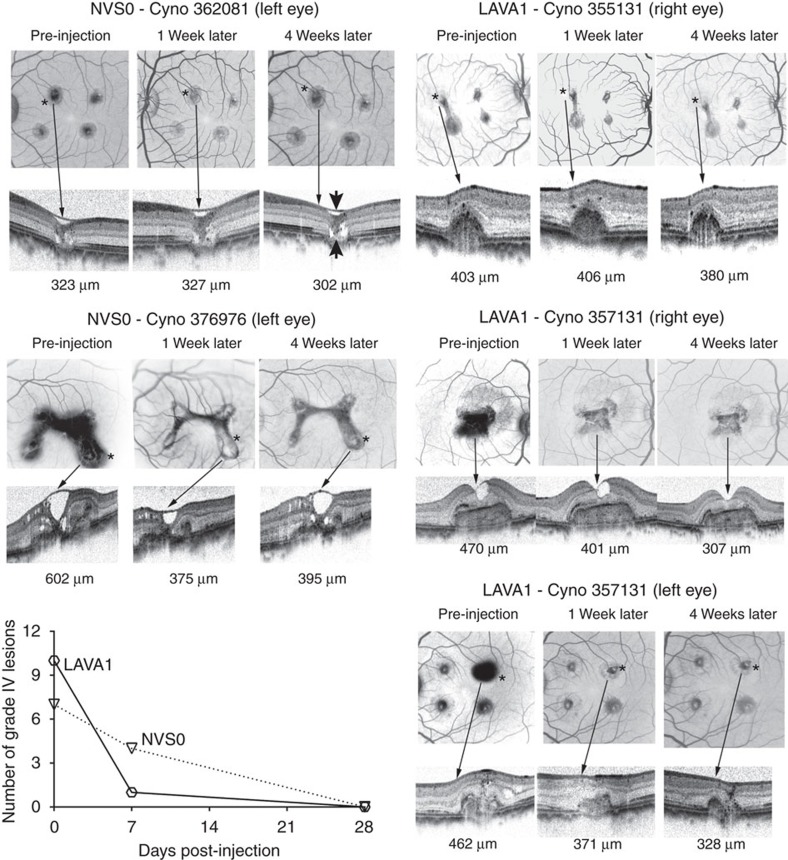
Efficacy of NVS0 and LAVA1 in the cynomolgus monkey laser-CNV model. CNV lesions were induced 4–9 months before dosing. Representative images of cynomolgus monkey eyes injected with NVS0 or LAVA1. The fundus images are shown as negatives (black and white reversed) to highlight the vascular leakage of fluorescein during fluorescein angiography. Each eye received four laser burns producing neovascular tufts (CNV) that appear as dark spots in the images; the darkness corresponds to the degree of vascular leakage from the lesions. The asterisks ‘*' in the fundus images correspond to the lesion from which retinal thickness was measured by optical coherence tomography (OCT). Total retinal thickness (values shown in the bottom of each OCT scan) was measured at the center of the lesion using calipers provided in the Spectralis software as the distance between the inner limiting membrane and retinal pigment epithelium (RPE) as shown by the arrow heads in the OCT of 362081 left eye. Both NVS0 and LAVA1 markedly reduced CNV-induced retinal oedema within 4 weeks. The plot shows the number of grade IV lesions at the time of dosing, 1 week and 4 weeks post dosing with NVS0 or LAVA1. Both NVS0 and LAVA1 reduced the number of grade IV lesions and retinal oedema within 4 weeks of injection.

The pharmacological activity of LAVA1 was assessed in two cynomolgus monkey studies using a laser-induced choroidal neovascularization (CNV) model, a commonly used animal model for neovascular AMD^14^. The first study was conducted in monkeys that had CNV induced by laser photocoagulation applied 4–9 months before the injection of NVS0 or LAVA1. Just before the injection of NVS0 or LAVA1, the laser-induced CNV lesions were documented to have vascular leakage by fluorescein angiography (Fig. 3). A total of three eyes in two animals received intravitreal injections of 200 μg (3,400 pmol) LAVA1 and two eyes in two animals received injections of 214 μg (4,400 pmol) of NVS0. Both LAVA1 and NVS0 reduced CNV-induced retinal oedema and leakage (Fig. 3). Slit-lamp examinations of the eyes 2, 7 and 28 days after injection revealed no significant ocular adverse events from either of the drugs.

**Figure 4:**
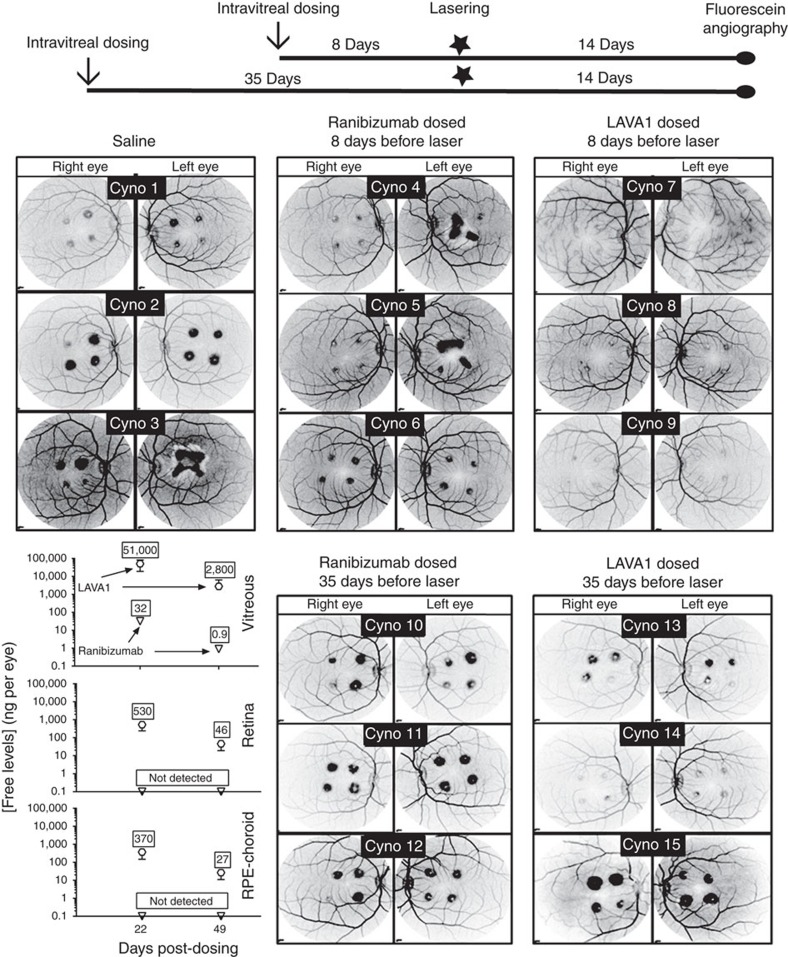
Efficacy of ranibizumab and LAVA1 in the cynomolgus monkey laser-CNV model. Laser photocoagulation to induce CNV lesions was applied 1–5 weeks after drugs were dosed. Late-phase fundus images from cynomolgus monkey eyes injected with ranibizumab or LAVA1 taken approximately 5 min post-intravenous fluorescein injection. The fundus images are shown as negatives (black and white reversed) to highlight the CNV-induced vascular leakage of fluorescein during fluorescein angiography. Cynomolgus monkeys 1–9 were imaged 22 days after dosing, whereas cynomolgus monkeys 10–15 were imaged 49 days after dosing. Each eye received four laser burns producing neovascular tufts (CNV) that appear as dark spots in the images; the darkness corresponds to the degree of vascular leakage from the CNV lesions. When dosed 8 days before laser, a single dose of ranibizumab only partially inhibited laser-induced CNV, whereas a single dose of LAVA1 completely inhibited laser-induced CNV. The plot (bottom left) shows average terminal levels of free (unbound to VEGF) LAVA1 and free ranibizumab in the cynomolgus monkey eyes 22 and 49 days post-intravitreal injection. Drug concentrations less than the lower limit of quantification (0.1 ng per eye) were categorized as ‘not detected'. At both day 22 and 49, free LAVA1 levels in the vitreous, retina and RPE-choroid were markedly higher than free ranibizumab. Error bars represent 1 s.d. Measurements were done in triplicates.

**Figure 5:**
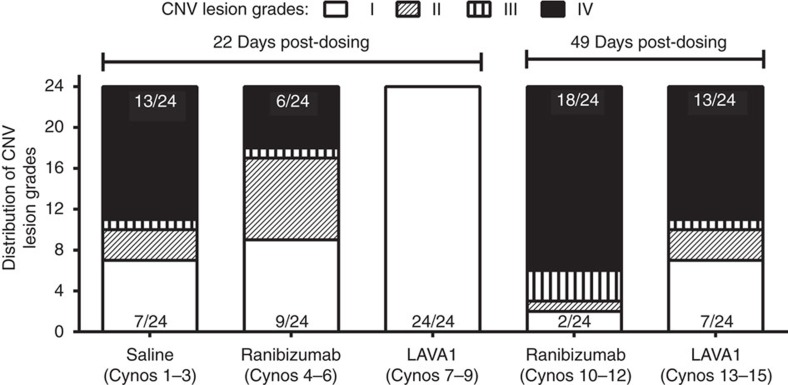
Distribution of average CNV lesion grades. In the cohort of monkeys that were imaged 22 days post dosing, eyes that received ranibizumab had six grade IV lesions, whereas none of the eyes that received LAVA1 had grade IV lesions (all lesions were grade I). In the cohort of monkeys that were imaged 49 days post dosing, eyes that received ranibizumab had 18 grade IV lesions, whereas eyes that received LAVA1 had 13 grade IV lesions.

The second cynomolgus laser-CNV study compared the duration of efficacy of LAVA1 with ranibizumab (Fig. 4). Cynomolgus monkeys (three animals per group=six eyes per group) received bilateral injections of equimolar doses (∼5,400 pmol) of ranibizumab (263 μg) or LAVA1 (324 μg) 8 or 35 days before laser photocoagulation to induce four CNV lesions per eye. Two weeks post laser, the lesions were evaluated by fluorescein angiography (Fig. 4). A bar graph of the proportions of lesions of each severity grade in each group is shown in Fig. 5. In the animals that received ranibizumab 8 days before laser, 6/24 lesions (cynomolgus monkeys 4–6 in Fig. 5) were at the most severe grade IV. In contrast, in the eyes that received LAVA1, none of the 24 laser burns were grade IV (cynomolgus monkeys 7–9 in Fig. 5). In the animals that were dosed 35 days before laser, 18/24 lesions (cynomolgus monkeys 10–12 in Fig. 5) were grade IV in the ranibizumab group, whereas only 13/24 lesions (cynomolgus monkeys 13–15 in Fig. 5) were grade IV in the LAVA1 group. The results of the monkey studies suggest that LAVA1 has a longer duration of action than ranibizumab.

Following the final fluorescein angiography assessments, animals were killed and drug concentrations were measured in the vitreous, retina, the retinal pigment epithelium-choroid and blood. Twenty-two days post dosing, the mean free drug levels of ranibizumab and LAVA1 in the vitreous were 32 ng per eye (653 fmol per eye or ∼0.012% of the injected dose) and 51 μg per eye (860 pmol per eye or ∼15.9% of the injected dose), respectively (Fig. 4). Forty-nine days post dosing, there was little or no detectable ranibizumab (lower limit of quantitation=0.1 ng per eye) in the vitreous, whereas there was a mean of about 2.8 μg per eye (47 pmol per eye or ∼0.9% of the injected dose) of LAVA1. LAVA1 was also detected in the retina and retinal pigment epithelium-choroid at both days 22 and 49 post dosing, whereas ranibizumab was not detected in these tissues (Fig. 4). LAVA1 was undetectable in the blood at any time point after dosing into the eye and ranbizumab was only detected in one animal 24 h post dosing. The presence of LAVA1 in the ocular tissues, as well as its ability to reduce the severity of laser-induced CNV lesions, demonstrates its reduced ocular clearance and its ability to diffuse into clinically relevant tissues at therapeutic amounts.

### Application of the HABP to non-VEGF proteins

To determine whether the HABP could be used to increase the ocular retention of different types of proteins including antibodies against targets besides VEGF, the HABP from hTSG6 was fused to the following proteins: an anti-erythropoietin Fab, an anti-TNFα Fab, an anti-properdin Fab, an anti-complement factor 5 Fab, an anti-VEGF IgG, an erythropoietin receptor (EPOR-Fc) trap, a VEGF receptor-1/2-Fc trap, an anti-VEGF designed ankyrin repeat protein (DARPin) and the hormone erythropoietin. The effect on vitreal half-life of fusing the HABP onto these proteins was determined by measuring terminal vitreal concentrations of the proteins with and without the hyaluronan-binding tag in rabbits 3 weeks after injection of 105 pmol per eye of each protein (Supplementary Table 1). In all cases, proteins fused with the hyaluronan-binding LINK domain had terminal concentrations that were 8- to 3,500-fold higher than the parent molecule or a molecule of the same structural category.

### Optimization of LAVA1

**Figure 6:**
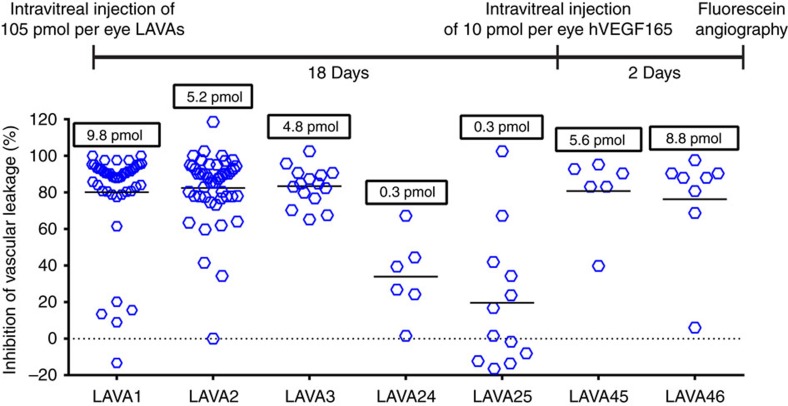
Efficacy of engineered variants of LAVA1 in the rabbit retinal vascular leakage model. Variants of LAVA1 that were engineered to be resistant to proteolysis (LAVA2, 3, 45 and 46) all potently inhibited fluorescein leakage and had terminal vitreal levels similar to LAVA1 when administered 20 days before fluorescein angiography. In contrast, non-glycosylated variants of LAVA1 (LAVA24 and LAVA25) minimally inhibited fluorescein leakage. The numbers in boxes above the datapoints are the mean terminal drug levels in the vitreous. LAVA24 and LAVA25 had terminal levels that were 39-fold lower than LAVA1. Terminal vitreal levels measured in ng ml^−1^ were converted to pmoles per eye using a molecular weight of 59 kDa for all LAVAs. Vitreal volume in rabbit was assumed to be 1.25 ml.

The human LINK domain sequence of TSG6 naturally has an N-linked glycosylation site. To reduce heterogeneity of the final product, we designed two variants (LAVA24 and LAVA25) of LAVA1 where this N-linked glycosylation site was deleted by amino acid substitutions of the asparagine residue. LAVA24 and LAVA25 had affinities for hVEGF and hyaluronan similar to LAVA1 (Supplementary Table 2 and Supplementary Data 2); however, both LAVA24 and LAVA25 had reduced efficacy in the rabbit VEGF-challenge model (Fig. 6), suggesting that the glycosylation of LAVA1 was important for *in vivo* activity possibly due to better *in vivo* stability.

During the expression and purification of LAVA1, proteolytic degradation products were observed by SDS–polyacrylamide gel electrophoresis, reverse-phase chromatography and mass spectrometry. The fraction of drug that was degraded ranged from 10% when expressed in HEK293 cells to 100% in certain CHO lines (see Supplementary Fig. 2 for a representative example). Mass spectrometry of material purified using an anti-Fab affinity resin revealed three distinct proteolytic cleavage sites, all located within the HABP. The proteolytic sites were adjacent to lysine and arginine residues that were exposed in unstructured loop regions and were either within or were in close proximity to hyaluronan-binding sites identified previously^15^. We created 55 new versions of LAVA1 with individual or combinations of amino acid substitutions around the observed digestion sites to search for a version that was resistant to proteolytic degradation and yet retained hyaluronan-binding activity. Most of the new versions were poorly expressed in HEK293 cells or had no hyaluronan-binding as determined by surface plasmon resonance affinity measurements (Supplementary Data 2). It was observed that substitutions of residues at or around the digestion sites alone did not completely eliminate proteolysis. Consequently, cysteine residues were introduced to mediate an additional disulfide bond in the LINK domain (as in LAVA45 and LAVA46) (Supplementary Data 2). Introduction of the additional disulfide bond along with amino acid substitutions at and around the observed digestion sites resulted in molecules that resisted proteolytic degradation and had hyaluronan-binding affinity similar to LAVA1 (Supplementary Data 2). The binding of LAVA2, 3, 24, 25, 45 and 46 for hVEGF-A_165_ was similar (Supplementary Table 2).

To determine whether the LAVAs with modified hyaluronan-binding domains affected the duration of action *in vivo*, the optimized LAVAs were assesed in the rabbit VEGF-challenge model (Fig. 6). Similar to LAVA1, the optimized, protease-resistant variants LAVA2, 3, 45 and 46 potently suppressed vascular leakage 20 days after intravitreal administration into rabbit eyes, indicating that the changes made in these molecules retained *in vivo* activity. Upon completion of fluorescein angiography, animals were killed and terminal drug levels were measured (Fig. 6). The average amount of LAVA2, 3, 45 and 46 remaining at day 21 ranged from 4.8 to 8.8 pmol per eye compared with 9.8 pmol per eye previously measured for LAVA1.

**Figure 7:**
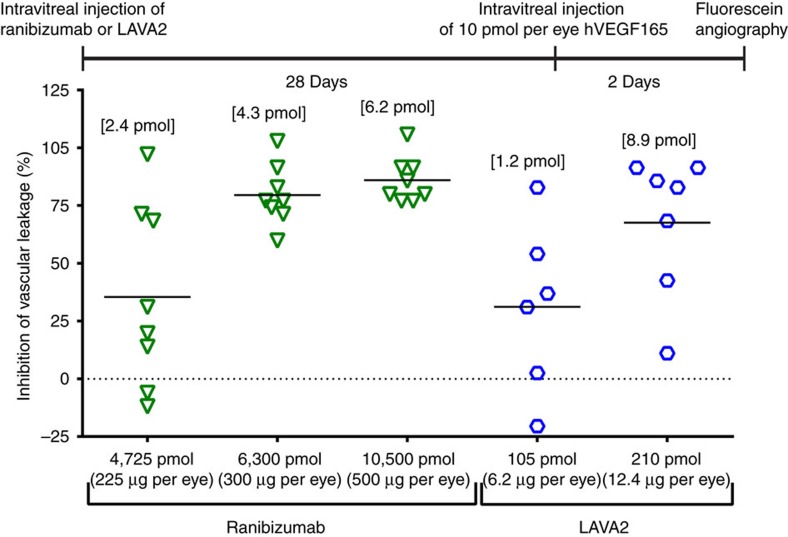
Efficacy of ranibizumab and LAVA2 in the rabbit retinal vascular leakage model. LAVA2 at a dose of 210 pmol demonstrated similar efficacy to ranibizumab doses of 6,300 or 10,500 pmol when administered intravitreally 30 days before fluorescein angiography. The terminal vitreal levels (values in brackets above the datapoints) of LAVA2 (8.9 pmol) are higher than the terminal vitreal levels of ranibizumab at all doses (2.4 to 6.2 pmol) even though ranibizumab doses were 22.5- to 50-fold higher than LAVA2 doses. Terminal vitreal levels measured in ng ml^−1^ were converted to pmoles per eye using a molecular weight of 49 kDa for ranibizumab and 59 kDa for LAVA2. Vitreal volume in rabbit was assumed to be 1.25 ml.

To further demonstrate the difference in ocular clearance rates and duration of efficacy between ranibizumab and a LAVA construct in the rabbit VEGF-challenge model, LAVA2 at doses of 6.2–12.4 μg (105–210 pmol) was compared with ranibizumab at doses of 225–500 μg (4.5–10 nanomoles). In molar terms, these doses of LAVA2 were 22 to 100-fold lower than the ranibizumab doses (Fig. 7). Thirty days post dosing, the 12.4 μg dose of LAVA2 demonstrated efficacy superior to a ranibizumab dose of 225 μg. Consistent with the observed efficacy, 31 days after a dose of 12.4 μg of LAVA2 the vitreous had 8.9 pmol of drug remaining (∼4.4% of injected dose), whereas 31 days after a 50-fold higher molar dose of ranibizumab (500 μg) there were only 6.2 pmol remaining (∼0.06% of injected dose). (Fig. 7). These data demonstrate that protein drugs that are fused with the HABP can be dosed at very low doses (as much as 50-fold lower doses) and still have comparable or superior activity as the parent unmodified drug.

## Discussion

This study describes protein drugs that have prolonged ocular retention after intravitreal administration. The half-life extension is conferred by a peptide that binds to hyaluronan, a major macomolecular structural component of the vitreous of the eye. We applied the method to both antibody and non-antibody types of drugs. Our most extensive characterization and optimization involved long-acting anti-VEGF antibodies, which we call LAVAs. The fusion of an optimized HABP to an anti-VEGF Fab had minimal impact on its anti-VEGF activities as determined by *in vitro* and *in vivo* assays including surface plasmon resonance (Biacore) affinity measurements, hVEGF-receptor-2 neutralization assays and two *in vivo* pre-clinical models. The biochemical and biophysical properties of the HABP were optimized by employing structural biology-directed protein engineering. Although there were some differences in the biochemical and biophysical properties of the variants of the HABPs, LAVAs that contained these variants generally demonstrated markedly longer duration of action compared with their foundation drugs.

Half-life extension of therapeutic proteins by binding to hyaluronan has advantages over other sustained delivery approaches such as biodegradable or non-biodegradable implants (Retisert, Ozurdex, Iluvien), encapsulated cell technology or implantable reserviors (ForSight, Replenish MicroPump), which require complicated manufacturing steps and surgical administration^16^,^17^. A therapeutic protein fused to an hyaluronan-binding peptide is readily designed and manufactured and can be delivered by a simple intravitreal injection.

Based on the increase in ocular half-life observed in rabbits and cynomolgus monkeys, it is expected that a LAVA molecule will enable a dosing frequency of not more than four times per year and probably without the need of any successive loading doses that some current standard-of-care anti-VEGF therapies require. The long-acting technology described here has the potential to be applied to other intra-ocular biologic therapies for ocular diseases including diabetic retinopathy, retinal vein occlusion, uveitis and glaucoma. This approach may also enable similar extension in local residence time in hyaluronan-rich compartments other than the vitreous, such as in synovial fluid in joints.

## Methods

### Materials

Aflibercept (Regeneron, Tarrytown, NY), Bevacizumab (Genentech/Roche, Basel, Switzerland) and Ranibizumab (Novartis, Basel, Switzerland, or Genentech/Roche) were used in this work. Human VEGF-A_165_ was procured from Peprotech (catalogue number AF-100-20). All other proteins were expressed and purified using methods described below.

### Mutagenesis and expression

The anti-VEGF Fab NVS0 was produced by converting the anti-VEGF scFv brolucizumab to a Fab format (Fig. 1). Hybrid proteins were produced by fusing LINK domains to the C terminus of proteins. For Fabs, the LINK domain was fused to the heavy chain, and for IgGs and Fc traps it was fused to the Fc region. Standard mutagenesis techniques were used to substitute residues of interest in the hyaluronan-binding LINK domain. Briefly, mutagenesis primers were designed and ordered from Integrated DNA Technologies. PCR mutagenesis was conducted using the QuickChange II mutagenesis kit (Agilent Technologies catalogue number 200521) as per the manufacturer's protocol. All plasmids were sequence-verified and transformed into *Escherichia coli* for scale-up DNA extraction. All proteins, except the DARPin, were expressed by transient HEK293 expression and purified by affinity purification using CaptureSelect resins from GE Healthcare as per the manufacturer's protocols. The DARPin was expressed and purified as described by Binz *et al*.^18^ All purified proteins were analytically characterized using SDS–PAGE, analytical size exclusion chromatography and mass spectrometry before use. Sites of proteolytic cleavage were identified using mass spectrometery. Binding of proteins to hVEGF or hyaluronan were determined either by ELISAs, ForteBio Octet or Biacore as per the manufacturer's protocol.

### Rabbit VEGF-challenge model

All *in vivo* studies were performed in accordance with procedures approved by the Novartis Institutional Animal Care and Use Committee. Male Dutch-belted rabbits (body weight 1.6–2.0 kg) were placed under general anaesthesia for both intravitreal injections and ocular imaging. Anaesthesia was induced with an intramuscular injection of ketamine and xylazine (17.5–35 and 2.5–5 mg kg^−1^, respectively) and corneas were desensitized with the topical application of 0.5% proparacaine. Rabbit eyes were dilated with topical 1% cyclopentolate and phenylephrine (usually 10% but occasionally 2.5% depending upon availability). Fifty microlitres of the test article, saline or a dose of hVEGF-A_165_ were injected superotemporally 2 mm inferior to the limbus into the vitreous with a sterile single-use 30-gauge needle under a surgical microscope. Immediately after injection, the rabbit eyes were examined directly with a surgical microscope for evidence of haemorrhage, retinal detachment, lens injury or regurgitation of the injected fluid. Subsequently, rabbits were administered a single topical dose of an antibiotic ointment (tobramycin) with or without corticosteroid (Tobrex or TobraDex). The Tobrex ointment was applied only after VEGF challenge doses. hVEGF-A_165_ for intravitreal injections was purchased from Peprotech (catalogue number AF-100-20) and reconstituted in 0.9% sterile sodium chloride solution.

Forty-eight hours after the hVEGF-A_165_ challenge, retinal vessel leakage was assessed with scanning laser ophthalmoscopy (SLO)-based fluorescence angiography. All retinal images were obtained with a 6-mode Spectralis camera (Heidelberg Engineering) equipped with a 30° lens centred on the nasal medullary ray adjacent to the optic nerve. Retinal vessels were labelled with an intravenous injection of 1 ml of 35–70 mg ml^−1^ 2,000 kDa fluorescein isothiocyanate (FITC)-dextran (Sigma catalogue number FD2000s) solution dissolved in sterile PBS. The concentration was chosen based on demonstration of clear labeling of the rabbit retinal vessels. The same concentration and dose of FITC-dextran was used for all rabbits in an experimental run. Retinal vessel permeability was subsequently assessed by bolus intravenous injection of 0.3 ml of 10% sodium fluorescein in the marginal ear vein. Images were acquired from the right eye at approximately 3 min (±15 s) and from the left eye at approximately 4–6 min after fluorescein injection. Data analysis was performed on retinal vessel images that were composed of an average of up to 40 registered individual SLO images. Animals were killed the same day or the day after completion of fluorescein angiography by an intravenous injection of 0.5 ml kg^−1^ Euthasol (Virbac, catalogue number 710101). Whole eyes were collected and snap-frozen in liquid nitrogen and stored at −80 °C.

Images were masked and randomized for the measurement of vascular leakage. Vascular permeability was quantified by processing the FITC-dextran image and corresponding fluorescein image acquired 48 h after the VEGF-A_165_ challenge, using a software routine developed in MATLAB. The dextran and fluorescein retinal images were first co-registered by either selecting several common landmarks manually or by using an image processing algorithm^19^. The optic nerve and area outside of the medullary ray were cropped out of the co-registered images, as well as any regions with insufficient image quality for analysis. The normalized, co-registered images were subsequently subtracted from each other on a pixel-by-pixel basis. The FITC-dextran image contained labelled retinal vessels, whereas the fluorescein image contained signal in the retinal vessels in addition to extravasated dye outside the vessels. Therefore, subtraction of the FITC-dextran image from the fluorescein image produced an image containing only the extravasated fluorescein. The resulting fluorescein intensity per unit area quantified from the subtracted image was reported as ‘fluorescein leakage' for that eye. Inhibition of vascular leakage was subsequently calculated for each eye relative to the average leakage observed in the control group that did not receive an anti-VEGF molecule. For example, eyes in test groups exhibiting leakage equivalent to the average value of the control group would yield an inhibition of vascular leakage of 0%. Inhibition was presented as either the mean value of the group (Fig. 2) or from individual eyes (Figs 6 and 7). Eyes were excluded from analysis based on observations of severe backflow of test article or VEGF-A_165_ noted at the time of injection, inflammation during or after an intravitreal injection (inflammatory cloudy debris), or poor image quality at the time of acquisition or during masked analysis.

### Cynomolgus laser CNV model

Non-human primates (*Macaca fascicularis*, weight from 2.4–5.8 kg) were used in these studies. All procedures were performed in accordance with and approved by the Alcon or Novartis Institutional Animal Care and Use Committee. Monkeys were sedated with an intramuscular injection of a combination of ketamine (5–20 mg kg^−1^), midazolam (0.05–0.5 mg kg^−1^) and glycopyrrolate (0.005 mg kg^−1^). Sedation was maintained with supplemental intravenous propofol (2–5 mg kg^−1^) as needed for up to 30 min. For imaging sessions, monkey eyes were dilated with topical phenylephrine (2.5%) and tropicamide (1%). Sedated animals were positioned with their eyes aligned with the optical pathway for laser photocoagulation, fundus photography and fluorescein angiography. At the conclusion of imaging, the monkeys were returned to their housing or were killed and their ocular tissues and plasma samples collected.

For laser photocoagulation to induce CNV, the sedated monkey's cornea was desensitized with topical proparacaine followed by placement of a 1 × Reichel Mainster contact lens (Ocular Instruments) with GenTeal Gel (Novartis) as the corneal lubricant. Four laser pulses were applied near the posterior pole of the eye equidistant from the fovea with a Novus Varia Three-Mode 657 nm Laser System (Lumenis). The laser was focused to obtain a spot size of approximately 75 μm, the laser pulses had a power of 600 mW and the pulse duration was 0.05–0.1 s. For intravitreal injections, monkey corneas were desensitized with topical proparacaine 0.5%. Fifty microlitres of the test article or saline were subsequently administered superotemporally into the vitreous using a single-use 29-gauge needle attached to a 0.3 ml Monoject syringe. Injections were performed under a surgical microscope using a magnifying contact lens to visualize the retina. Immediately after an injection, one drop of moxifloxacin 0.5% was administered as well as a topical dose of atropine 1% ointment.

A Spectralis system was used to acquire both optical coherence tomography (OCT) (with a 30° lens) and fluorescein angiography images (30° or 55° lens). For OCT, a 13-line, 5 × 15° scan grid was positioned over each CNV lesion to document the appearance in cross section. The mean retinal thickness (the distance in micrometres measured from the retinal pigment epithelium to the inner limiting membrane) was measured for each lesion. A scan pattern centred on the lesions at the first time point after laser was set as the reference for follow-up imaging. For fluorescein angiography, a 1.0 ml bolus of 5% sodium fluorescein was injected through an intravenous port in the saphenous vein. Early phase filling was captured as a movie (approximately 0–20 s after injection). A late-phase image (acquired as an average of up to 30 SLO images) was taken approximately 5 min after intravenous injection of fluorescein. Four masked observers graded the late phase images of CNV lesions using a four point subjective scoring system^20^. The average severity levels of all four scorers for each lesion was averaged and categorized as Grade I when the average score was 1–1.4, Grade II when the average score was 1.5–2.4, Grade III when the average score was 2.5–3.4 and Grade IV when the average score was 3.5–4.0.

### Terminal drug measurements

Animals were killed via a systemic injection of sodium pentobarbital under deep anaesthesia and whole eyes were enucleated. Enucleated eye balls were trimmed by removing remaining connective tissues and muscles. Eyes were then immediately snap frozen by immersing in liquid nitrogen. Frozen eyes were stored at −80 °C until further use. On the day of dissection, a small incision was made at the limbus and continued around the circumference of the eye to separate the anterior and posterior chambers. Four small 4–6 mm incisions in the anterior portion of the posterior globe were made at the superior, inferior, lateral and medial sides. The whole, frozen vitreous humor was then collected. The posterior eye cup was then transferred in a petridish and placed under the microscope. The retina surface was gently wiped with a microspear sponge to remove remnants of vitreous humor. Entire retina was gently peeled off and collected in a pre-weighed microcentrifuge tube. The RPE-choroid was collected using a Gill knife and placed in a pre-weighed microcentrifuge tube. All tubes were identified based on treatment group, animal number, ocular tissue, collection time point and sample number. The weight of the tissues was recorded for all ocular tissues using an analytical balance. Samples were immediately placed on dry ice after weighing, followed by storage at −80 °C for bioanalysis. Ocular tissues such as vitreous humor, retina and retinal pigment epithelium dissected from frozen eyes were homogenized at 4 °C using a Tissue Lyzer (Qiagen, Inc.) as per the manufacturer's protocol by applying a vibration frequency of 20 Hz for 2 min at intervals of 2 min for a total of three to five cycles. Homogenized samples were centrifuged at 15,000 *g* and the supernatant was collected and used for measurement of terminal concentrations of molecules used in the study.

### ELISAs for rabbit vitreal terminal concentrations

Sandwich ELISAs were performed by coating Maxisorp 384-well plates (Nunc catalogue number 464718) with 20 μl of a goat anti-human IgG (H+L) (Thermo Fisher catalogue number 31119) in carbonate buffer (Pierce catalogue number 28382) overnight at 4 °C, then washed three times with Tris-buffered saline (TBS) containing Tween-20 (Thermo Scientific catalogue number 28360) using a BioTek plate washer. Plates were blocked for 2 h at room temperature (or overnight at 4 °C) with blocking buffer containing 2 or 5% bovine serum albumin (Sigma catalogue number A4503 or Roche catalogue number 03116964001), 0.1% Tween-20 (Sigma catalogue number P1379), 0.1% Triton X-100 (Sigma catalogue number P234729) in TBS, followed by washing three times for 5 min each in TBS containing Tween-20. Ocular tissue samples were diluted in TBS containing 2% bovine serum albumin (Sigma catalogue number A4503), 0.1% Tween-20 (Sigma catalogue number P1379) and 0.1% Triton X-100 (Sigma catalogue number P234729). Samples were then incubated on the coated plate for 1 h at room temperature with gentle shaking. Plates were then washed three times for 5 min each time and incubated for 30 min at room temperature with gentle shaking, with the detection antibody (goat anti-human IgG [F(Ab′)2]) conjugated to horseradish peroxidase (Thermo Fisher catalogue number 31414)). After washing, the chromogenic substrate tetramethylbenzidine (Thermo Fisher catalogue number 34028) was added for 15 min, following which the reaction was quenched with 2 N sulfuric acid (Ricca catalogue number 8,310–32). The absorbance of the samples were read on a spectrophotometer at 450 nm and 570 nm (blank subtraction) as per the manufacturer's instructions. For each test article, purified proteins were used to generate 12-point standard curves from starting concentrations of 200 ng ml^−1^ with twofold dilutions to interpolate protein recovery levels from ocular tissues. Data were fit using four-parameter non-linear regression in GraphPad Prism. Unknown sample concentrations were interpolated from the standard curve (*Y*=Bottom+(Top−Bottom)/(1+10^(LogEC_50-*X*_)^ × HillSlope)) and then multiplied by the dilution factor of the sample in order to determine the final concentration recovered.

### Gyrolab assay for cynomolgus ocular terminal concentrations

Terminal concentrations of ranibizumab and LAVA1 in ocular tissues were measured using the Gyrolab miniaturized high-sensitivity immunoassay method (Gyros, Inc., Warren, NJ). Before analyses, tissue samples were thawed at room temperature for 5 min. Vitreal samples were diluted in Rexxip AN Max buffer (Gyros AB, Inc. catalogue number P0004995) for a final dilution of 1:2 in a 96-well plate (Thermo Scientific catalogue number AB-800). Samples were sealed with microplate covers (Gyros AB, Inc., catalogue number P0003313) and mixed thoroughly in a plate shaker for 1 min. After ensuring that there were no bubbles in the bottom of the wells, the samples were measured in a Bioaffy1,000 CD in the Gyrolab xP workstation. A 3-step C-A-D method was executed on the Gyrolab xP workstation; capture antibody was flowed through the system first, followed by the analyte (the sample) and then the detector antibody. The Gyrolab xP workstation performed washes of PBS containing 0.01% Tween-20 (Calbiochem, Inc. catalogue number 655206) in between each step. The standard curve was prepared using a starting concentration of 12,000 ng ml^−1^ in matrix (BioReclamation, LLC., catalogue number Rabb-Vitreous) followed by sixfold serial dilutions to a final concentration of 0.3 ng ml^−1^.

Retina and RPE-choroid samples were diluted in Rexxip AN Max (Gyros AB, Inc. catalogue number P0004995) for a final dilution of 1:2 in a 96-well plate (Thermo Scientific catalogue number AB-800). Samples were sealed with microplate covers (Gyros AB, Inc. catalogue number P0003313) and mixed thoroughly in a plate shaker for 1 min. After ensuring that there were no bubbles in the bottom of the wells, the samples were measured in a Bioaffy200 CD in the Gyrolab xP workstation. A three-step C–A–D method was executed on the Gyrolab xP workstation; capture antibody was flowed through the system first, followed by the analyte (samples) and then the detector antibody. The Gyrolab xP workstation performs washes of PBS containing 0.01% Tween-20 (Calbiochem, Inc. catalogue number 655206) in between each step. The standard curve was prepared using a starting concentration of 30,000 ng ml^−1^ in matrix (naive cynomolgus retina and RPE-choroid) followed by sixfold serial dilutions to a final concentration of 0.6 ng ml^−1^. Free drug was measured by applying 100 μg ml^−1^ biotin-labelled hVEGF-A_165_ (Peprotech catalogue number AF-100-20) to a column containing streptavidin-coated particles. Samples were applied to activated columns in a parallel manner, one sample per column. Free drug was captured by biotinylated hVEGF-A_165_ and retained on the column. Proteins not retained on the column, including the paired hVEGF-A_165_-Fab complex, were washed away in the wash step. Subsequently, for detection, a solution of 25 nM Alexafluor-647 labelled goat anti-human IgG antibody (Bethyl Laboratories, catalogue number A80-319A) in Rexxip F buffer was applied to each column. Visualization and measurement were achieved by laser-induced fluorescence detection at 647 nm. Alexafluor labelling was performed using Life Technologies labelling kit (catalogue number A-20186) as per the manufacturer's protocol. The capture reagent was prepared in PBS containing 0.01% Tween-20 and the detector reagent in Rexxip F (Gyros AB, Inc., catalogue number P0004825). The data generated from GyroLab was imported to SoftMax Pro v5.4.1 (Molecular Devices). A five-parameter fit was applied to derive the correlation between concentration and the response value from GyroLab. Subsequently, the correlation equation was used to calculate concentrations for the samples from their response values.

To calculate the lower limit of detection (LLOD) the following definition was used in this report.


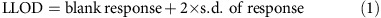


The s.d. of response and the signal-to-noise were directly obtained from the GyroLab instrument. SoftMax Pro was used to calculate the concentration corresponding to the response values relating to the LLOD.

### Data availability

The authors declare that the data supporting the findings of this study are available within the paper and its Supplementary Information files.

## Additional information

**How to cite this article:** Ghosh, J. G. *et al*. Long-acting protein drugs for the treatment of ocular diseases. *Nat. Commun.* **8,** 14837 doi: 10.1038/ncomms14837 (2017).

**Publisher's note**: Springer Nature remains neutral with regard to jurisdictional claims in published maps and institutional affiliations.

## Supplementary Material

Supplementary Information Supplementary Figures and Tables.

Supplementary Data 1 Expression and characterization of an anti-VEGF Fab fused with various HA-binding domains.

Supplementary Data 2 Sequence of LAVA 1 and locations of amino acid substitutions made to generate additional LAVA1 variants.

## Figures and Tables

**Table 1:** Terminal vitreal levels of anti-VEGF molecules from the rabbit leakage model.

**Test Article**	**Average vitreal concentrations (pmol per eye)**					
	**At day 20**	**At day 21**	**At day 24**	**At day 26**	**At day 30**	**At day 31**
Brolucizumab	—	0.004	Not performed			
Ranibizumab	—	0.1	Not performed			
Aflibercept	—	2.1	0.1	Not performed		
Bevacizumab	—	1.2	Not performed			
NVS0	0.6	Not performed				
LAVA1	—	9.8	5.6	3.9	3.1	3.0

VEGF, vascular endothelial growth factor.

Terminal vitreal levels measured in ng ml^−1^ were converted to pmol per eye using the molecular weights of each molecule as follows: 26 kDa for brolucizumab, 49 kDa for ranibizumab, 97 kDa for aflibercept, 150 kDa for bevacizumab, 47 kDa for NVS0 and 59 kDa for LAVA1. Vitreal volume in rabbit was assumed to be 1.25 ml. Data shown are from several independent studies.
