# Supplementary material for: Long-acting protein drugs for the treatment of ocular diseases
Source: Nat Commun. 2017 Mar 23;8:14837. doi: 10.1038/ncomms14837 (PMC5376645; doi:10.1038/ncomms14837)
Supplement: Supplementary Information — Supplementary Figures and Tables. [file ncomms14837-s1.pdf]

Supplementary Figures

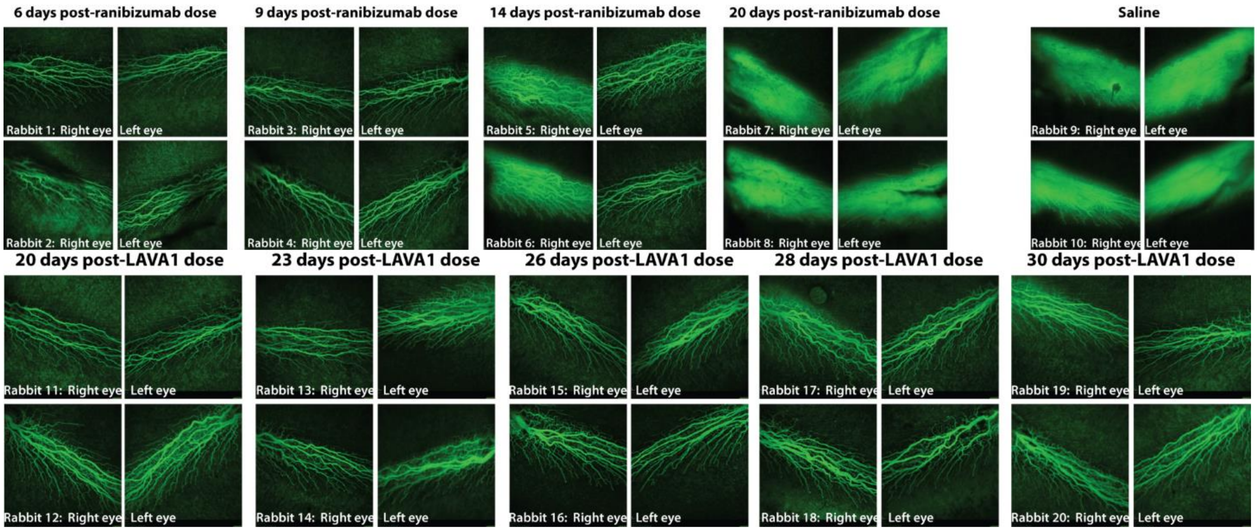

Supplementary Figure 1: Comparison of the duration of action of ranibizumab and LAVA1 in the rabbit hVEGF-A_{165} challenge model. Rabbit eyes were injected with 400 ng (10 pmoles) hVEGF-A_{165} to induce retinal vascular permeability. Forty-eight hours later, fluorescein angiography was performed to measure the leakage of fluorescein from retinal vessels. Representative right and left eye images from two rabbits from each group are shown. Retinal vessels without leakage have fluorescence only inside the vessels due to labeling by high molecular weight fluorescein-dextran, while leaky blood vessels have fluorescein both within their lumens as well as diffusely outside. Ranibizumab inhibited retinal vascular leakage when dosed up to 9 days before fluorescein angiography but not when dosed 14 or 20 days before fluorescein angiography. In contrast, LAVA1 completely inhibited retinal vascular leakage for up to 30 days (longest time duration assessed).

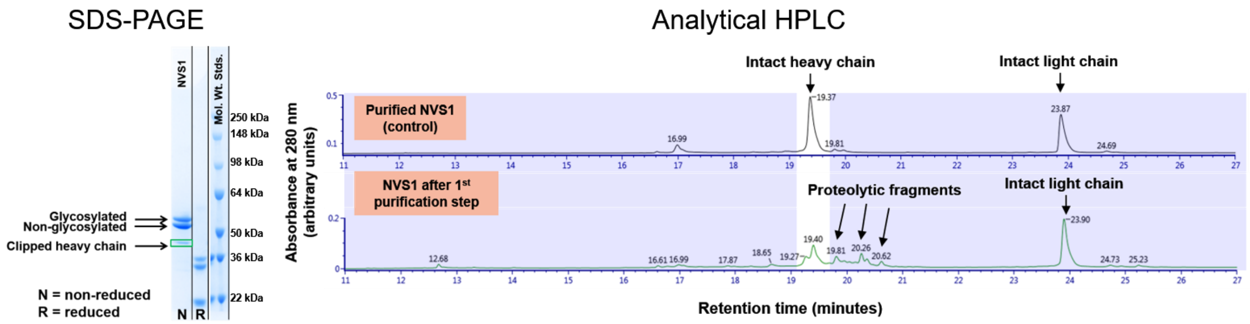

Supplementary Figure 2: Biochemical characterization of the integrity of NVS1. Left panel: SDS-PAGE analysis of affinity-resin purified NVS1 after transient expression in HEK293 cells. Right panel:Analytical HPLC analysis of affinity-resin purified NVS1 purified after stable expression in CHO cells. In both cell expressions systems, the heavy chain of NVS1 was proteolytically degraded at various sites in the LINK domain.

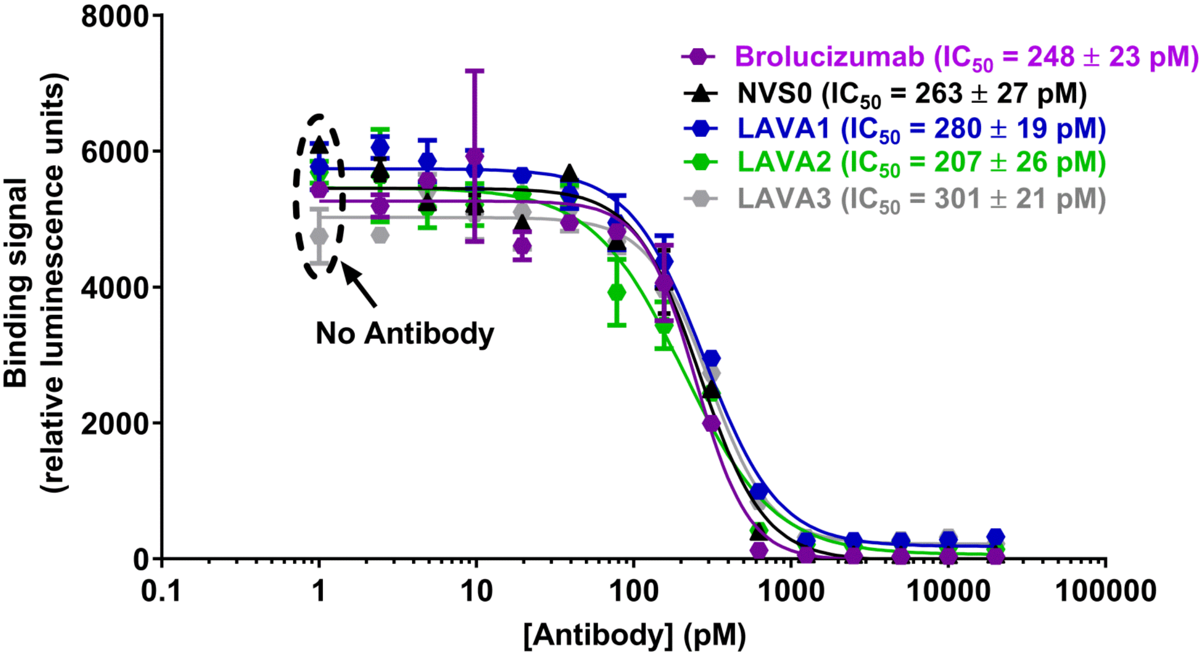

Supplementary Figure 3: Inhibition of binding of hVEGF-A_{165} to human VEGFR2-Fc. All LAVAs block binding of hVEGF-A_{165} to hVEGFR2-Fc with IC_{50} values ranging from 207-301 pM, similar to the untagged anti-VEGF Fab, NVS0. IC_{50} values are provided with standard errors derived from fits to the data. Error bars represent one standard deviation. Measurements were done in triplicates.

Supplementary Tables

Supplementary Table 1: Terminal vitreal concentrations and calculated 2-point ocular half-life ($t_{1/2}$) values. Drug concentrations that were below LLOQ of 20 ng/ml were assigned a value of 20 ng/ml to calculate $t_{1/2}$ values.

NVS ID	Target	Format	Injected dose (ng)	Terminal vitreal conc. (ng/ml)	2-point ocular $t_{1/2}$ (days)	Terminal vitreal conc. by mass spectrometry (ng/ml)	2-point ocular $t_{1/2}$ (days)
NVS70T	C5	Fab	6200	710	6.7	792	7.1
NVS71T	Properdin	Fab	6200	432	5.5	607	6.2
NVS73T	TNFα	Fab	6200	212	4.3	223	4.4
NVS77T	FGFR2	Fab	6200	357	5.1	2553	16.5
NVS76T	IL-17A	Fab	6200	92.5	3.5	466	5.6
NVS72T	EPO	Fab	6200	Not done		108	3.6
NVS74T	Factor D	Fab	6200	Not done		969	7.9
NVS90T	EPOR	Hormone	3000	145	1.2	Not done	
NVS78T	EPO	Fc Trap	13500	267	3.7	Not done	
NVS81T	VEGF	IgG	7500	1039	7.4	189	4
NVS82T	VEGF	IgG	7500	414	5	122	3.5
NVS80T	VEGF	Fc Trap	12400	1346	6.6	Not done	
**Molecules without HA-binding peptide tag**							
NVS77	FGFR2	Fab	5000	0.1	1.4	20	2.6
NVS73	TNFα	Fab	5000	12	2.4	20	2.6
NVS90	EPOR	Hormone	3000	17.2	0.5	Not done	
NVS81	VEGF	IgG	7500	15	2.3	20	2.5
NVS82	VEGF	IgG	7500	13	2.3	20	2.5
NVS80	VEGF	Fc Trap	10000	11	2.1	Not done	

Supplementary Table 2: Kinetic rate constants and affinity of LAVA constructs for biotinylated hVEGF-A_{165} and biotinylated 17 kDa hyaluronan.

	Binding to 17 kDa hyaluronan			Binding to hVEGF-A_{165}		
**Molecule**	**ka (M^-1*s^-1)**	**kd (s^-1)**	**K_D (M)**	**ka (M^-1*s^-1)**	**kd (s^-1)**	**K_D (M)**
NVS0	No binding			5.51E+06	7.87E-05	1.42E-11
LAVA1	7.21E+05	1.33E-01	1.85E-07	5.75E+07	1.01E-05	1.76E-13
LAVA2	4.34E+05	3.59E-01	8.27E-07	4.10E+07	1.85E-04	4.53E-12
LAVA3	6.23E+05	1.42E-01	2.28E-07	7.39E+07	1.19E-05	1.61E-13
LAVA24	1.35E+05	4.34E-01	3.21E-06	2.25E+07	1.98E-04	8.82E-12
LAVA25	1.22E+06	9.21E-01	7.55E-07	1.86E+07	2.13E-04	1.15E-11
LAVA45	3.91E+06	3.63E-01	9.28E-08	2.35E+07	7.21E-05	3.07E-12
LAVA46	2.52E+06	3.69E-01	1.46E-07	6.23E+07	2.57E-04	4.13E-12
